# Correction: Implementation of good clinical practice in clinical research in the context of limited resources settings: Lessons learnt from the freeBILy trial using an embedded mixed methods approach

**DOI:** 10.1371/journal.pntd.0014087

**Published:** 2026-03-10

**Authors:** Leonard Gunga, Pia Rausche, Rivo Andry Rakotoarivelo, Mandranto Rasamoelina, Jeannine Solonirina, Elveric Fesia, Ravo Razafindrakoto, Njary Rakotozandrindrainy, Mickael Radomanana, Valentina Marchese, Nagham Issa, Jean-Marc Kutz, Aaron Remkes, Anna Jaeger, Dewi Ismajani Puradiredja, Govert van Dam, Norbert Schwarz, Jürgen May, Raphaël Rakotozandrindrainy, Natalie Fischer, Daniela Fusco, on behalf of the freeBILy consortium

The 14th and 15th authors’ name are spelled incorrectly. The correct name is: Anna Jaeger and Dewi Ismajani Puradiredja.
