# Supplementary material for: Implementation of good clinical practice in clinical research in the context of limited resources settings: Lessons learnt from the freeBILy trial using an embedded mixed methods approach
Source: PLoS Negl Trop Dis. 2026 Feb 9;20(2):e0013899. doi: 10.1371/journal.pntd.0013899 (PMC12900435; doi:10.1371/journal.pntd.0013899)
Supplement: S1 Table — (DOCX) [file pntd.0013899.s001.docx]

**S1 Table : Overview normalized data entry errors by timepoint**

| **Study Visit** | **Total of Incorrect Data Entry Fields,**  **N** | **Number of Data Entry Fields,**  **Median (IQR)** | **Incorrect Data Normalized by Data Entry Fields,**  **(%)** |
| --- | --- | --- | --- |
| **T0** | 909 | 58 (56 - 61) | 3.1 |
| **T1** | 594 | 33 (31 - 35) | 3.6 |
| **T2** | 768 | 39 (9 - 40) | 3.9 |
| **T3** | 1529 | 114 (11 - 127) | 2.7 |
| **T4** | 1350 | 98 (85 - 126) | 2.8 |
| **Other*^1^*** | 61 | 9 (8 - 10) | 1.4 |
| *^1^*Other represents the study withdrawals. | | | |
