# Supplementary material for: Implementation of good clinical practice in clinical research in the context of limited resources settings: Lessons learnt from the freeBILy trial using an embedded mixed methods approach
Source: PLoS Negl Trop Dis. 2026 Feb 9;20(2):e0013899. doi: 10.1371/journal.pntd.0013899 (PMC12900435; doi:10.1371/journal.pntd.0013899)
Supplement: S2 Table — (DOCX) [file pntd.0013899.s002.docx]

**S2 Table: Incorrect data entry fields by type of variable**

| **Incorrect data entry fields** | **N** | **n (%)** |
| --- | --- | --- |
| **Variable type** | 5,211 |  |
| **Date** |  | 1,299 (24.9) |
| **Binary categorical** |  | 1,249 (24.0) |
| **Text** |  | 1,053 (20.2) |
| **Categorical with levels** |  | 1,048 (20.1) |
| **Numeric** |  | 543 (10.4) |
| **Study ID** |  | 19 (0.36) |
