# Supplementary material for: Implementation of good clinical practice in clinical research in the context of limited resources settings: Lessons learnt from the freeBILy trial using an embedded mixed methods approach
Source: PLoS Negl Trop Dis. 2026 Feb 9;20(2):e0013899. doi: 10.1371/journal.pntd.0013899 (PMC12900435; doi:10.1371/journal.pntd.0013899)
Supplement: S3 Table — (DOCX) [file pntd.0013899.s003.docx]

**S3 Table: Incorrect data entry fields and outcome variables**

| **Number of incorrect primary outcome data entry fields,**  **n** | **Total number of primary outcome variables,**  **N** | **Incorrect data entry fields,**  **(%)** |
| --- | --- | --- |
| 123 | 10,798 | 1.1 |
