# Supplementary material for: Implementation of good clinical practice in clinical research in the context of limited resources settings: Lessons learnt from the freeBILy trial using an embedded mixed methods approach
Source: PLoS Negl Trop Dis. 2026 Feb 9;20(2):e0013899. doi: 10.1371/journal.pntd.0013899 (PMC12900435; doi:10.1371/journal.pntd.0013899)
Supplement: S4 Table — (DOCX) [file pntd.0013899.s004.docx]

**S4 Table: Study staff characteristics**

|  | **Staff Members** | **Median (IQR)** |
| --- | --- | --- |
| **Age in Years** | 30 | 31 (29, 34) |
|  |  |  |
|  |  | **N (%)** |
| **Sex** | 30 |  |
| Female |  | 23 (76.7%) |
| Male |  | 7 (23.3%) |
| **Main Contributor to Household Income** | 30 |  |
| freeBILy staff |  | 10 (33.3%) |
| Partner |  | 1 (3.3%) |
| Relative |  | 18 (60.0%) |
| Other |  | 1 (3.3%) |
| **Additional Job** | 30 |  |
| Yes |  | 4 (13.3%) |
| No |  | 26 (86.7%) |
| **Regular Reading in French** | 29 |  |
| Yes |  | 25 (86.2%) |
| No |  | 4 (13.8%) |
| Unknown |  | 1 |
| **Highest Level of Education** | 30 |  |
| University |  | 30 (100.0%) |
| **Self-Assessment of French Skills** | 30 |  |
| Fluently writing in French on a professional level |  | 6 (20.0%) |
| 4 |  | 10 (33.3%) |
| 3 |  | 11 (36.7%) |
| 2 |  | 3 (10.0%) |
| Trouble writing short messages in French |  | 0 (0.0%) |
| **GCP Trainings Attended in Lifetime** | 30 |  |
| 1 |  | 1 (3.3%) |
| 2 |  | 8 (26.7%) |
| 3 |  | 5 (16.7%) |
| 4 |  | 10 (33.3%) |
| 5 |  | 5 (16.7%) |
| 10 |  | 1 (3.3%) |
| **Participation at a GCP Training in the last 2 Years** | 30 |  |
| Yes |  | 29 (96.7%) |
| No |  | 1 (3.3%) |
| **Start Working in the freeBILy team** | 30 |  |
| Mar 2018 |  | 2 (6.7%) |
| Oct 2018 |  | 1 (3.3%) |
| Nov 2018 |  | 19 (63.3%) |
| Dec 2018 |  | 5 (16.7%) |
| Aug 2019 |  | 2 (6.7%) |
| May 2020 |  | 1 (3.3%) |
