# Supplementary material for: Implementation of good clinical practice in clinical research in the context of limited resources settings: Lessons learnt from the freeBILy trial using an embedded mixed methods approach
Source: PLoS Negl Trop Dis. 2026 Feb 9;20(2):e0013899. doi: 10.1371/journal.pntd.0013899 (PMC12900435; doi:10.1371/journal.pntd.0013899)
Supplement: S5 Table — (DOCX) [file pntd.0013899.s005.docx]

**S5 Table: Framework matrix**

| **N= 30** | **Number of participants** | **Example Quotes** |
| --- | --- | --- |
| Themes | **n** |  |
| **Understanding of GCP** |  |  |
| GCP ensures the reliability of the trial | 3 | Good clinical practices are rules laid down to ensure the reliability of a clinical trial - *ID 12, Data Entry* |
| GCP is easily understandable | 3 | Good practice was easy to understand - *ID 05, Midwife* |
| Confidentiality and Secrecy are important | 10 | In research, it was very important to respect secrecy and respect between the two parties - *ID 01, Nurse* |
| GCP is the signature of the consent | 5 | That's part of good clinical practice, you have to respect the participant's wishes, whether she wants to take part in the study or not. That's why we have informed consent, so that the participant gives her consent for the study and so that - *ID 15, Data entry* |
| GCP is about the participant | 5 | What I remember first when we talk about good clinical practice is ensuring the health of the people we care for - *ID 04, Paramedic* |
| Quality control is important | 5 | We'd have to follow the procedures, because there was […] a risk of getting a false result and, above all, there was also a risk of not reassuring the participant if we didn't carry out the GCP - *ID 01, Nurse* |
| GCP is following a set standard | 11 | Good clinical practice, as I understand it, is always, there are steps that you have to follow. We follow each step one by one, and we must ensure that a step is not missed for it to be good clinical practice. If one of these steps is incomplete, it is no longer good clinical practice at that point- *ID 10, Midwife* |
| **Attitude towards GCP** |  |  |
| Did not study for exam | 1 | there wasn't much preparation as they say, because I've already experienced that during my daily work, but not to do a training course - *ID 10, Midwife* |
| Studied for the exam | 3 | Yes, as an exam you had to be prepared. It wasn't anything new yet, but it was something we'd already experienced in our day-to-day work. It was something that we've already practised on a daily basis but some things were new and it helps in the work too - *ID 13, Lab-Technician* |
| Experienced non- compliance with GCP in the study | 5 | PIDs and TIDs don't match up sometimes. And there were times when I was picking it up because about the samples for example, and maybe it was difficult especially for the children's samples, it was difficult to pick it up but there was something that didn't fit, and it was in the laboratory that we found it that we couldn't use because it didn't follow the normal pattern, and it was lost - *ID 13, Lab-Technician*  Good clinical practice was not respected because there are a lot of patients and the staff are in a hurry to finish the work, and this was not respected - *ID 05, Midwife* |
| Following GCP is improving study results | 5 | Because it improves the work, the quality of the work and the result - *ID 14, Student data entry* |
| Compliance with GCP in the study | 6 | But we always followed the training we did here. The training we received we applied - *ID 01, Nurse* |
| Positive perception of GCP | 10 | Good clinical practice is, it helps me a lot, especially when it comes to clinical trials - *ID 03, Midwife* |
| **Informed consent** |  |  |
| Withdrawls mid study | 3 | I don't know if it was 3 or 4 withdrawals, who didn't want to continue, but theirs was at the beginning that the people there knew that we took blood, and they said that we didn't do it anymore, and that was the difficulty - *ID 06, Midwife* |
| Filling IC is time consuming | 3 | We always found ways of getting consent, we had to find ways of convincing the person but we couldn't stay like that, we had to explain even if we wasted time, sometimes we wasted a lot of time explaining things to someone - *ID 04, Paramedic* |
| Difficulties and misconceptions implementing the IC | 7 | A lot of them were asking questions, because as soon as you start with consent, there were already questions about why you had to sign it, and now they're already worried that there might be rumours, for example, about child trafficking - *ID 05, Midwife* |
| Special groups were respected | 6 | What was important in the consent process, first of all, was that it was explained to her, that she clearly understood what we should do to her, and, once she had understood, we asked her to sign, as an adult, who knew how to read. If she was illiterate, we needed a witness who could read and write. If she was a minor, she needed a guardian to sign. If she was a minor and illiterate, she needed a witness and a guardian - *ID 04, Paramedic* |
| IC is important | 2 | What was important in all this was the participants' knowledge of the activities to be carried out on them - *ID 01, Nurse* |
| **Workflow** |  |  |
| Quality control is time consuming | 3 | And there again, perhaps I'm in favour of the person asking me that quality control is a time delay and I'm in favour of it too, perhaps we can minimise it in our study - *ID 15, Data entry,* |
| Routine improved workflow | 8 | At first it was difficult because I'm not used to it, but as the work progressed, I got used to it - *ID 04, Paramedic* |
| Documentation as time consuming factor | 8 | And there was also the problem of time, for me particularly, it there was a lot of paperwork to fill in- *ID 30* |
| High workload | 5 | Yes, there were times, it took a long time, sometimes we didn't have lunch, because we'd have to follow it, but at the end we'd share it, the questionnaires were added up - *ID 05, Midwife* |
| **Barriers implementing GCP** |  |  |
| Study procedures and IC changed mid study | 4 | The second small problem for me, I didn't know about the other people, but it seemed that today we're doing something A and tomorrow it will change to B - *ID 17, Nurse* |
| Insufficient infrastructure | 1 | We had to take the mother's weight, and freeBILy didn't have an adult scale and that made a traffic jam at the CPN but it made the freeBILy office and the CPN office far apart. And that meant that we had to go back and forth to weigh a person - *ID 18, Paramedic* |
| Unclear quality control results | 2 | There were times when, when we did it, we couldn't see the control clearly and then we repeated it again - *ID 12, Data Entry, Antananariv* |
| Sub-studies | 2 | often in a research maybe there are sub studies, and when there was, no, just do it, but at the beginning you have to make it known that in clinical research there have to be sub-studies, but there wasn't that at the beginning, but when it arrived the work multiplied but there was no motivation*- ID 19, Data Entry* |
| Time pressure | 7 | It was the time that seemed, um, we're trying to manage the time because there were a lot of people but respecting consent we have to take people one by one - *ID 10, Midwife* |
| Loss of GCP knowledge over project duration | 1 | But maybe, for example, urine sampling and all that for example, I'd suddenly forgotten to do it*- ID 02, Office worker* |
| Inability to track participants | 6 | It was this time alone that was the real problem, because if people left and maybe the next time they didn't come back- *ID 24, Nurse*  the most difficult I've seen, because our T4, we made an effort to, because she had to make the five visits, which we thought at the beginning, and that we could find them all. If we had to take them from the CSB, we'd have to go down to each fokontany - *ID 10, Midwife* |
| Fear of participants | 5 | what they thought of the signing was that the Vazaha took the children after they had given birth - *ID 05, Midwife*  in T0 we need to take blood samples, pee and poo samples and at the eighth month visit it will just be a follow-up of this. And it was a bit, many were the people we did like that, people were afraid of it in our village *ID 30* |
| COVID-19 Impacted recruitment | 1 | When it had spread too much because there were a lot of cases of corona in Miarinarivo*, - Office worker* |
| **Facilitators implementing GCP** |  |  |
| Quality control | 2 | In my opinion, quality control was useful because in this quality control, we already knew what was going on *- ID 16, Nurse* |
| Study documents helped implementing GCP | 1 | In fact, it wasn't really difficult because we've already had the training, and the SOPs help a lot, and I could see that it wasn't serious but everything was going well - *ID 10, Midwife* |
| GCP training of the staff | 3 | For me, in good practice, for example, in a clinical study, it's always useful to do training on good practice because to be able to manage the study well - *ID 15, Data entry*  when I started working here, there was good training, and it was often and I think it gave coherence to my work - *ID 01, Nurse* |
| Travel expenses paid for participants | 2 | I think we should give it, even if it's only for travel expenses, because some participants come from very far away, only for the expenses we have to pay- *ID 07* |
| No incentives given to participants | 2 | And they didn't have to be given money to be motivated, according to the standard -  *ID 01* |
| **Motivation to work in a GCP based trial** |  |  |
| Research interest | 3 | I was very interested if it was about bilharziasis because it was in the hospital and it concerned the hospital again, this bilharziasis, and I went in and also to develop knowledge*- ID 23, Community Worker* |
| Convenience | 10 | The reason I'm still working in freeBILY, because the job search was even harder. And then I didn't want to change jobs - *ID 12, Data Entry* |
| Monetary gain | 5 | Like, I joined the project primarily for the money. My thesis wasn't done yet and for the preparation, I don't know, it still needed money. And that was just the reason for joining, but there was no particular reason to do a clinical study or anything like that. - *ID 15, Data entry* |
| Autonomy in working time | 3 | Because the project has time, we have time to do things, for example, we work for half a day and in the afternoon I can do my own private work - *ID 05, Midwife* |
| Extend professional skillset | 9 | Yes, it's in the project that I've found it's much better to do a clinical study. Before, all I was thinking about was money, but when I joined the project, I found that it could be a career, I found good times, good training, and I got to meet ex-Principals like you. And finally, maybe if things go too far, I'll go and study abroad, where I'll be able to see the world - *ID 15, Data entry* |
| **Recommendations to facilitate GCP adherence** |  |  |
| Adapt GCP rules to the local context | 2 | I don't know if there will be any research in the future, professors and doctors in Madagascar will get together to discuss what good practice is more compatible here? because it's not the same, for example, abroad, if we talk about health and they're happy, but here at h o m e even taking a step is money, time and all that*- ID 19, Data Entry* |
|  |  |  |
| Give incentive to participants | 5 | Yes, but some of the participants still say that the 2000ar is smaller for them, maybe they need to increase it a bit - *ID 15, Data entry*  In good clinical practice it should be that you should not give money, but we are Malagasy, for us a thing is done for free it has no value for people, and this was one of his motivations to participate even if they received coffee - *ID 17, Nurse* |
| Improve study material | 3 | In the check-list there was the address of the participant and it must be precise, a precise address, for example, we put the lot of the house and all that because it was complicated for the CAs to look for the participants afterwards*- ID 16, Nurse* |
| Improve communication | 4 | Mine, about good clinical practice, that was there, but if I understand the training correctly from that time on we had already defined who all the participants in this study were, and I apologise to the "vazaha" but I didn't understand them any more, every day there were new vazaha arriving, what did they all do? and if they arrived here they gave us tasks and these tasks that they asked us to do we didn't know the end, I apologise for the registers who they were?- *ID 17, Nurse* |
| More training for all staff | 5 | My wish about improving work like this is about training, we need, everyone has their own way of receiving, and we need, it depends on being able to repeat it - *ID 13, Lab-Technician* |
| Optimize time consuming activities | 5 | Making an effort, for example, shortening activities, which is all I wanted to say - *ID 01, Nurse* |
| Employ more qualified staff | 2 | But there are midwives who are capable of it and there are midwives and nurses with more of an explanation maybe it doesn't get into their heads or whatever. And here too I'm going to ask an idea about the recruitment of midwives maybe we need to have experience with midwives before recruiting them or something like that so that we don't have people who don't even understand French or something like that- *ID 15, Data entry* |
|  |  |  |
