# Supplementary material for: Implementation of good clinical practice in clinical research in the context of limited resources settings: Lessons learnt from the freeBILy trial using an embedded mixed methods approach
Source: PLoS Negl Trop Dis. 2026 Feb 9;20(2):e0013899. doi: 10.1371/journal.pntd.0013899 (PMC12900435; doi:10.1371/journal.pntd.0013899)
Supplement: S6 Table — (DOCX) [file pntd.0013899.s006.docx]

S6 Table: Charting of quantitative and qualitative finding for triangulation

| **Elements of quality in low-resource clinical trails** | **Quantitative findings** | **Qualitative findings** | **Output** |
| --- | --- | --- | --- |
| **Building factors** |  |  | Main building factor is the collection and validation of reliable trial data, facilitated through GCP reporting guidelines and the training and re-training of employees. Capacity strengthening is a vital element. However, the collection of the data also comes with a high workload in documentation for the staff which need to be constantly assessed to mitigate negative effects on the staff and the trial quality. |
| Participant safety | In 12.8 % (64/ 500) of ICs, an error (i.e., use of the older form) had been corrected | Importance of consent process for vulnerable groups heavily emphasized |  |
| Adherence to guidelines | Errors within the acceptable range, 96.7% of staff attended multiple GCP trainings in their life | Following guidelines and SOP were named as key elements, though changes of these documents during trial made adherence challenging |  |
| Patient centeredness | Not measured | Protecting confidentiality of participants was reported across all professions, community engagement activities mentioned positively, though adding workload |  |
| Soundness | Reapproval of ethics committee due to changes in study, re-training was implemented (Fig. 2) | Procedures in the handling of vulnerable groups were followed |  |
| Documentation | 0.6 % the name of the nurse obtaining IC missing, and the date of the nurse's signature missing in 5.2 %, most commonly dates (e.g. date of vaccination) were wrongly documented | Strong workload of documentation on all levels of the trial needed to be done, (In healthcare centre, laboratory and data entry), ambivalent opinions about routine, one said it impacted negatively, most staff stated routine facilitated proper documentation |  |
| Data integrity | Improvement of documentation with routine (z = -6.968, Mann-Kendall Test, p < 0.001), Low proportion of severe or moderate errors in data entry fields (1.5%)  Primary outcome 1.1% of entry field incorrect | Not mentioned |  |
| **Promoting factors** |  |  | Establishing trial structures, be it team structure, infrastructure or structures to manage community engagement are seen as promoting factors. It is vital to for the sustainability of these structures, also after trial end, that they are build in partnership with local stakeholders and agencies |
| Context adaptation | 13.3% of employees had another job besides the trial, all staff had a university degree | Long driving times to reach the participants, adaptation of GCP for the Malagasy context mentioned with the reference to the importance of reimbursement of costs that emerge due to trail participation, improving communication with study participants was recommended |  |
| Infrastructure | Not measured | Infrastructure: Tools sometimes needed to be fetched from other buildings as not available,  Team infrastructure: Differing levels of French made communication within the staff challenging |  |
| Partnerships | Not measured | Sustainable research structures emphasized |  |
| Quality system | Logging records traceable, all changes in database were recorded and retrievable | Quality control understood as a key component of GCP, however it was also seen as adding huge workload, sometimes quality ensuring measures were not functioning (e.g. non-compliant labelling of samples leading to discard). Routine improved the implementation of quality control |  |
| Operational excellence | Not measured | Efficiency of documentation can be improved, waste of sampling material due to mislabeling and close expiration dates of diagnostic material need monitoring |  |
| **Emerging:**  **Assessment factors** |  |  | Additional to the previous categories of the framework, factors requiring assessment in trial planning and conduct have been identified, these factors to mainly focus on the robustness of a trial and the interaction of social responsibility and trial implementation. The identified factors are in close interplay with the two previous domains of the framework |
| External threats (Covid-19) | Covid-19 impacted data collection heavily (grey shaded areas Fig. 2), second monitoring only remote possible due to COVID-19 lockdown | Staff was fearful to get infected with Covid-19 when handling study visits *(Intertwined with attitudes and perceptions*) |  |
| Attitudes and perceptions towards the trial (Of participants and staff directed towards each other/ the trial) | Not measured | Rumors about the trial emerged before recruitment, staff needed to invest into counselling and careful explanation of trial procedures, women withdrew before blood drawing from the trail, assumingly due to fear, frequently rotating staff from study sponsor made communication of staff and study sponsor personal complicated, |  |
