# Supplementary material for: Implementation of good clinical practice in clinical research in the context of limited resources settings: Lessons learnt from the freeBILy trial using an embedded mixed methods approach
Source: PLoS Negl Trop Dis. 2026 Feb 9;20(2):e0013899. doi: 10.1371/journal.pntd.0013899 (PMC12900435; doi:10.1371/journal.pntd.0013899)
Supplement: Attachment — Submitted filename: Gunga et al, Response to reviewers.pdf [file pntd.0013899.s009.pdf]

BNITM
Bernhard Nocht Institute for Tropical Medicine

Bernhard Nocht Institute for Tropical Medicine
P.O. Box 50 06 40, 22706 Hamburg, Germany

Dr Daniela Fusco
Group leader

Bernhard-Nocht-Str. 74
20359 Hamburg | Germany
Tel +49 40 42818-504
Fax +49 40 42818-243
fusco@bnitm.de

Hamburg,09.12.2025

To the Editorial Board of PLOS Neglected Tropical Diseases

Dear Editor, dear reviewers,

Thank you very much for the positive feedback on our publication, we appreciate the interest in the topic which can support the advancement of clinical trials much needed in the field of neglected tropical diseases and diseases of poverty.

As required, we are uploading two versions of the manuscript: one clean and one with track changes.

We hope we correctly interpreted all requests and provided point by point comments to them. Please, if anything is unclear or consequence of misinterpretation of the comments, don't hesitate to come back to us for further clarifications.

Faithfully
Daniela Fusco, PhD

Bernhard Nocht Institute for Tropical Medicine • Foundation under Public Law • A member of the Leibniz Association
National Reference Centre for Tropical Pathogens
WHO Collaborating Centre for Arbovirus and Haemorrhagic Fever Reference and Research
Board of Directors: Prof. Dr. Jürgen May (Chair), Prof. Dr. Stephan Günther, Prof. Dr. Iris Bruchhaus, Birgit Müller
Chair of the Board of Trustees: State Counsellor Dr. Eva Gümbel
www.bnitm.de

PNTD-D-25-01527
Implementation of good clinical practice in clinical research in the context of limited resources settings: lessons learnt from the freeBILy trial using a multi-methods approach

Reviewer #1

- **Justify the sample size and assess representativeness by comparing the sample to the full cohort.**

Reply: We thank the reviewer for the comment. The sample size of 500 participants (±10% of the total study population) was based on literature undertaking similar analysis (e.g., Mealer et al. 2013 ane Houston et al. 2015, as stated in the manuscript). We assume that by using a random seed to draw a sample the subset is representative of the study population, without executing a comparative analysis. Some additional clarifying language regarding the sampling proceadures was added to the methods section.

Informed Consent (IC)

- **Adopt objective criteria to classify invalid IC and specify the analytical impact (e.g., exclusion in the primary analysis and inclusion in a sensitivity analysis). Align the discussion with current guidelines (e.g., ICH-E6(R3).**

Reply: Thanks a lot for this comment. To clarify, in the present analysis the participants' ID represents exclusively the unit for randomization. All following analysis performed with the data, represented quality analysis of the database entries and logging data, but not of the participants. The dataset is represented by the type of entry in the data base classified as critical, moderate or minor towards the main endpoint of the trial. Hence, no data was excluded from the analysis and no sensitivity analysis was conducted.
ICs were classified as invalid if any of it had missing participant/guardian/witness or nurse printed name or signature, as well as missing signature date, or missing thumbprint for illiterate participants. As additional information, for the main analysis of the study, not topic for this specific manuscript, the data from particpants with invalid IC was excluded.
In the discussion, adjustments were included in lines 548-549, 558-559, 565-569.

Triangulation and Integration

- **Include a summary table linking quantitative and qualitative findings to operational recommendations (who is responsible, when actions occur, and feasibility/cost).**

Reply: We thank the reviewer for this comment. The summary table of the quantitative and qualitative findings, linked over the operational recommendations which was the basis of this visual triangulation has been added to the supplementary material (Supplementary table 6). However, this charting only links quantitative and qualitative findings over the topics of the base framework to identify emerging concepts against this framework, further feasibility and costs cannot be determined at this point as the cost effectiveness component of the trial has not been analyzed, yet. We added the operational recommendation from the triangulation to the initial charting.

Language Review American English

Reviewer #2: The overall study design must be stated rest minor modifications in methods section.

Reply: We thank the reviewer for this comment. The overall study design has now been stated as an embedded mixed method. In accordance with guidelines this has additionally been integrated into the title of the manuscript.

Reviewer #3:
-Are the objectives of the study clearly articulated with a clear testable hypothesis stated?
= the objectives are clearly stated in lines 99-107

However, I am uncertain if data entry errors would be considered GCP non-compliance? Wouldn't data corrections (corrected appropriately) indicate adherence to GCP?

Reply: We thank the reviewer for this comment and we fully agree that data correction would clash with the concept of non-compliance. The intention of the study is indeed to demonstrate how we can improve data handling in order to favour GCP compliance and avoid massive errors that might affect the overall quality of studies. We hope that this clarifies.

== Lines 214-218: It would be nice to include more details on how you approached the triangulation
==Line 411: Could the authors elaborate more on the triangulation results? This seems to me to be the keystone to the work (bringing together two aspects of the analysis), but I struggle to conceptualize the results.

Reply: We thank the reviewer for this comment. The matrix charting building the base and approach towards this triangulation based on de Pretto Lazarova et al. has been added to the supplementary of this publication and is described in the method section in line 262-264.

==Line 160: "the results from the manually reviewed informed consent forms" – but it is unclear who manually reviewed them?

Reply: We thank the reviewer for this comment. The ICs were reviewed by the responsible research assistant in the field. Clarification has been added to the methods section.

== Line 166 and 167: The authors have not yet introduced the term "ISIs" and "FGD", I would recommend providing the full name here in the first instance (unless the list of abbreviations is meant to solely address this?)

Reply: We thank the reviewer for this comment. This inaccuracy has been corrected and ISI and FGDs were abbreviated in their first-time usage.

==Line 167: it's unclear what authors mean by "15 purposively selected employees for information rich cases" ? & Lines 167-169: Could the authors elaborate on why 15 staff were chosen for one set of interviews and 15 were chosen for the other? Were they randomly assigned? and what was the difference between the two interviews?

Reply: We thank the reviewer for this comment. The decision process on who to sample for the ISIs and FGDs was based on previous knowledge of the interview conducting researcher on a) Degree of involvement of the employees in trial procedures, b) dynamic of the participant in interview situations and c) group constellation of the FGDs to ensure an, as far as possible, balanced group dynamic. The

differences in the types of interviews can be found in a) the depth of individual exploration of topics, b) level of social desirability in the in-depth interview vs. group discussion among colleagues (though social desirability against the interviewer cannot be fully mitigated even in a confidential in-depth interview) and c) the dynamic of the group discussion which lead to an increased number of emerging themes in contrast to an in-depth exploration of a priori topics and themes in the semi-structured interviews.

Missing data entry fields, multiple data entries
== Lines 185-187: were missing fields considered as data entry errors?
==Lines 185-187: If double data entry was conducted, were the records merged in REDCap and then the errors analyzed? Or were the errors considered per data entry staff, for example?

Reply: We thank the reviewer for these comments. Analysis of data entry corrections were conducted on the merged dataset after double data entry. No analysis per data entry staff was conducted. Missing data entries were considered data entry errors only if they were subsequently corrected (i.e., modified from missing to complete). Regular REDCap data quality checks performed by the data manager minimized missing data in critical variables. Additional language was added to the methods section for more clarity (lines 416-419).

-

==Lines 194: Perhaps the authors would like to clarify "consents" (i.e. 500 consents were chosen)?

Reply: We thank the reviewer for this comment. "Consents" refers to "informed consent forms". Throughout the manuscript informed consent was abbreviated to "IC" for consistency, after the first occurrence.

-Is the sample size sufficient to ensure adequate power to address the hypothesis being tested?
==I do not see a problem with the sample size, but just a few comments here:
== Line 182: 500 participants, out of 4612 participants, should be 10.8% of the total number of participants, not 11.2%

Reply: We thank the reviewer for the comment. The total number of unique participants in our analytic dataset was 4,613. Of these, 500 participants were included in the subsample (500/4613 = 10.8 %). We applied a proportional sampling fraction of 11.2% within each PHCC group to ensure that: all PHCCs were represented and the relative contribution of each PHCC to the total sample was preserved. Because sampling was conducted independently within each group, with rounding at the PHCC level, the final total did not equal exactly 11.2%. This procedure resulted in exactly 500 participants, corresponding to 10.8% overall. Additional language was added to the methods section for more clarity (lines 413-416).

== Line 182: participants were "randomly selected" but could the authors elaborate on how this was done?

Reply: We thank the reviewer for the comment. Sampling and analysis was conducted using R 4.3.1. A seed was set and 11.2 % of participants were randomly sampled within PHCC strata, ensuring proportional representation of each PHCC. Additional language has been added to the methods section for clarification (lines 413-416).

Reviewer #2: Some calculations has to be redone as mentioned in the reviewer attachment.Table 1 needs to be redone with correct calculations.

Reply: We thank the reviewer for the comment. We assessed the calculations and numbers represented in table 1, but did not find any error. It might be that the rounding we adopted in the calculations led to different final numbers that nevertheless don't change the meaning of the results.

==Line 290: how did the authors decide which IC errors were minor or major?
-Are the results clearly and completely presented?

Reply: We thank the reviewer for the comment. IC errors in our study were not grouped into "minor" or "major" categories. Could it be possible that you are referring to the word "minor" as in regards to the age group? We did have "minors" participante in the freeBILy study. All IC errors were considered equally important.

==The authors may want to elaborate on how / if the study team changed throughout time, and if this had an impact on data quality

Reply: We thank the reviewer for this comment. During the 4 years of the FreeBILy trail, there ws limited turnover of local study staff (eg. research associated, nurses, data clerks), but the international sponsor staff (eg. study coordinator, student researchers) changed more frequently. The aspect of the rotating staff of the study sponsor was thematized in the FGDs and recommendations have been made towards this aspect. We did not evaluate any impact of change in sponsor staff on data quality in our study. However, monitoring visits to the field were conducted by all sponsor staff in a regular manner and we don't expect an impact of changes in staff in this regard.

==Lines 281: It would be helpful to know if the IC errors were corrected & how?

Reply: We thank the reviewer for this comment. Yes, invalid IC were re-obtained during regular study visits or upon visits to participants who had been informed by the community health workers. A new informed consent form was signed.

Original comments of the reviewer #2

Abstract

1) Abstract has to be rewritten as background-gap-objective-method-expected outcome to maintain flow; presently, the abstract ends at the objective.

Reply: we thank a lot the reviewer for the comment - there might have been possibly some problem with the documents received? The abstract is structured in Background, Methods, Results and Conclusions and it definitely doesn't end with the objectives.
We are pasting below the current version of the abstract to make sure that this can be properly visualised.

Background:
Limited financial and human resources and infrastructure can affect the implementation of Good Clinical Practice (GCP), which can have a detrimental impact on data quality and the robustness and application of clinical trial outcomes. Monitoring frameworks are designed to ensure good data quality and help to guide adaptations of trial procedures over time. However, these frameworks tend to be based on datacentric approaches, which often neglect vital aspects of trials, such as social responsibility, capacity strengthening, and contextual influence. Therefore, this study analyses barriers and facilitators of the implementation of GCP in resource-limited settings to inform the establishment of adapted frameworks for trial management and monitoring.

Methods:
In this multi-method analysis of the freeBILy trial, conducted in Madagascar from 2019-2022, a random subset of trial participants (n=500) and informed consents (n= 500) was analyzed for protocol deviations through descriptive statistics and trend analysis. Framework analysis of focus group discussions and individual semi-structures interviews provided a sociological viewpoint of the study context. Findings were subsequently triangulated, merging the viewpoints on the influences towards GCP in resource-limited settings.
Results:
A decreasing trend in incorrect database entries was found ($z = -6.968$, Mann-Kendall Test, $p < 0.001$) over the course of the study, with an overall rate of 1.8 % incorrect data entries. Triangulation showed three key areas of GCP implementation in resource-limited settings, which extend previous frameworks: a) Context adaptation towards infra-, team- and social structures as promoting factors, b) External influences, such as external threats, study personnel attitudes and perception towards the trial require recurrent assessment, and c) Promote GCP-compliant data collection subject to regular documentation and training cycles to facilitate capacity strenghtning and data ownership.
Conclusion:
This study shows the limitations of datacentric clinical trial management to assess GCP performances in the frame of clinical trials in resource-limited settings. We highlight the importance of well-trained and integrated study staff, as well as thorough preparation, budgeting and context appropriate monitoring. This achieves high quality, patient centered and compliant research, implemented through alternative frameworks for monitoring and evaluation.

2) The phrase "*adapted frameworks for trial management and monitoring"* needs clarity regarding what kind of adaptations are envisioned (e.g., community engagement, staff training, ethical oversight)

Reply: this point is addressed with the previous reply.

Introduction

3) The introduction does a good job of highlighting the significance of implementing GCP in low-resource situations, but it is far too long; it would be better to condense the repeating arguments.

Reply: thanks a lot for the comment. The background has been adapted as requested.

4) Although the term "diseases of poverty" is appropriate, it would be clearer if it were accompanied by concrete examples and figures.
5)

Reply: thanks a lot for the comment. Clarifications and refrrence have been added in lines 67-68.

6) Some sentences are too long and make it difficult to comprehend; shortening them might help readers understand them better.

Reply: thanks a lot for the comment. The text has been revised to improve clarity.

7) Although the explanation of ICH-GCP origins is instructive, it may be summarized sooner to keep the flow intact.

Reply: we thank the reviewer for the comment. We are not sure we fully understand this comment as the clarification of the ICH-GCP origins is reported in teh very first lines of the background. It might be that we didn't fully understand the comment, please add more clarifications if actions are required.

8) Statements on GCP's lack of contextualization are made repeatedly; to minimize repetition, think about combining them.

Reply: The text has been revised accordingly.

9) The paper should explain how social responsibility and capacity building were operationalized in the study, even though it appropriately emphasizes these points.

Reply: clarifications have been added as requested, in lines 122-124 (... During the trial measures of community engagement such as awareness initiatives and community involving side events such as the distribution of coffe plants were held to facilitate attrition of the trial...).

10) Although crucial, the term "contextualization" is not well defined; provide it with an operational definition with latest reference.

Reply: The term contextualisation has been further defined and a source discussing the influence of this factor onto clinical trials has been cited (Haenssgen et al., 2019, lines 87-89).

11) Although the claim of "high paperwork burden" is true, it requires citation or documentary proof.

Reply: Thanks a lot for this comment, we provided suitable citation for this aspect in the introduction (Tinto et al. 2013, line 119). In the results this aspect is heavily emphasised in the staff inetrviews.

12) The phrase should be standardized because "resource-limited" and "low-resource" are used inconsistently.

Reply: The phrasing has been adjusted and the term resource-limited settings has been used.

13) Provide a brief description of why Madagascar and the freeBILy trial were chosen as a case study.

Reply: Adjustments have been made in lines 124-127

Materials and Methods

14) While objectives (i) and (ii) are quantitative, (iii) is qualitative; specifically, state the overall study design.

Reply Thanks a lot for this suggestion, the overall study design has been stated in line 127-133 and integrated into the title.

15) In the methods section, line no.159, clarify if it's **form** or **from**?

Reply This section has been changed, this was a typo and from is correct

Results

16) In line 234, change the comma in "1,8%" to a period so that it reads 1.8%.

Reply: the value has been adapted as requested

17) In the same line 234, please change 1.8% of 294,477 to 5,300 instead of 5,211.

Reply: Thank you very much for this comment, the percentages are rounded to one decimal, taking the exact estimates, we confirm that 5211 is correct.

18) Please recheck the values of Table 1, in the column for incorrect data entries.

Reply: This connects to the comment above, the values are rounded to one decimal. We added a footnote to the table for clarification.

19) Line no. 287 and 288, please put a space after the slash.

Reply: Adjusted as requested (Now line 328)

20) Please avoid repeating sentences from the Materials and methods and starting a sentence with a numeric value.

Reply: Thanks a lot for these suggestions, this has been adapted

21) Please avoid repeating sentences from the Materials and methods and starting a sentence with a numeric value.

Reply: Thanks a lot for these suggestions, this has been adapted

22) In line 438, consider adding a space between and required.

Reply: Thanks a lot for this remark, it was adapted

23) Paper flow can be improved.

Reply: Thanks a lot for this comment. Reapeating passages have been removed and flow was revised hoping to have improved it.

Author's Contribution Section

24) In the author's contribution section, line no. 589, consider adding space between of and the.

Reply: adjusted as required.

References

25) Many of the references are lacking volume number, issue number, and page number. Please strictly adhere to the journal reference style.

Reply: thanks a lot for the comment. The references have been checked and adapted to the journal requirements now reported in the syle required by PLOS NTD. The modifications are not visible as track changes due to formatting constrains of word and the used citation manger.

Reviewer #3: -Are the conclusions supported by the data presented?
==The authors' conclusion is that interpreting datacentric results in the context of GCP is an insufficient approach in resource-limited settings. The authors indicate that training and context appropriate monitoring is paramount to ensure high quality and compliant research. However, I find it difficult to see succinctly how these conclusions were made. Perhaps if the authors could elaborate on the triangulation aspect of the results, it would become clearer. The data entry errors were very small overall, despite limited resources and extensive paperwork.

Reply: Thank you for this comment. Indeed, we found that our overall rate of incorrect data entry was small. However, despite training efforts, extensive paperwork contributed to critical errors, especially with regards to informed consents as seen in the quantitative analysis. For instance, the administrative workload reduced the time available to focus on these pivotal components of good clinical practice, as transpired in the FGDs. Future studies should consider to carefully balance between the amount of data collection and the extensive paperwork. Additionally, the employees voiced during FGDs how important training and routine was in reducing their errors made during data collection. In our quantitative analysis we found that the errors reduced correspondingly over time, even though the CFRs became longer with more data entry fields. This shows that repeaterd training over time strenghtens capacity and competency and hence leads to less errors made.

==Line 72: It could be helpful to provide a reference for E6 R2

Reply: We thank the reviewer for this comment. The E6 (R2) has been referenced in the introduction section of this manuscript as reference number 2.

==Line 81: Elaborating on how E6(R3) has shifted clinical trial management into the right direction could guide readers towards what are still limiting factors

Reply: We thank the reviewer for this comment. We added the for our publication vital information of stakeholder involvement in clinical trial planning (E6 (R3) (3.1.3) and the reference (Number 8) to the intertwinement with the E8 (R1) to the background to understand the context of our analysis better. Overall, our aim is to provide contextual information towards the implementation of the new E6 (R3) of leassons learned under the E6 (R2), therefore this manuscript is limited in providing a full analysis of the R3 beyond key aspects (e.g. fit for purposeness).

==Line 138: REDCap requests specific citations when their database is used (additional information located on their website under "Resources --> citations"). Authors should consider including this (https://doi.org/10.1016/j.jbi.2008.08.010 and https://doi.org/10.1016/j.jbi.2019.103208).

Reply: We thank the reviewer for this note. The proposed citations have been added to the manuscript as referneces number 16 and 17.

==Line 234: A full-stop should be used instead of a comma when reporting percentages (i.e. 1.8%)

Reply: We thank the reviewer for this comment. This has been reviewed and adapted through the manuscript.

==I am not sure if it's because of the 'justify' centering of the text, but there are a few instances where it appears there are 2 spaces between words (e.g. Line 274, 279, 216, 395)

Reply: We thank the reviewer for this comment. This has been reviewed and adapted through the manuscript.

==Line 338: I believe the authors meant to write 'midwives'

Reply: We thank the reviewer for this comment. This has been corrected in the text.

Summary

Sampling and Bias
Please provide detailed information on the randomization of the subsets: sampling design, the software used (and the random seed), and a step-by-step description of the selection procedure. Justify the sample size and assess representativeness by comparing the sample to the full cohort.

Reply: We thank the reviewer for this comment. The randomization procedure for the CRF data entry fields and IC has been explained in more detail in the manuscript including utilize R® packages (line 198 – 201, line 217 – 219). A justification for the sample size has been provided in response to a similar comment above.

Definitions and Error Metrics
Please clarify the operational definitions: what constitutes a final discrepancy between the CRF and the source? How are mandatory fields handled when missing?

I recommend adding a severity classification (critical/major/minor) and stratifying errors by variable type and timepoint.

Reply: Thank you for this comment. A severity classification (critical/severe/minor) was performed at all different study time points. No significant differences were found between the time points. This graph and analysis were not included in the final manuscript.

It would be interesting to compare, perhaps, deviations related to inclusion/exclusion criteria, prohibited medications, etc., and how / if the frequency of these deviations changed after training sessions or monitoring visits.

Reply: We thank the reviewer for this comment. Regular data checks in REDCap were performed regulary by the data manager of the study sponsor throughout the study to reduce inconsistent inclusion data (e.g., participants recorded as nine months pregnant at recruitment). For the purpose of this manuscript „inclusion criteria" fall under „major error" for the CRF at study timepoint 1. Incorrect data entries related to the primary outcome -pivotal fort the study's value- have been analyzed separately (see Supplementary Table 3).

In the same vein, it would be helpful if the authors compared how these results might differ from clinical trials in other resource-sufficient settings.

Reply: We thank the reviewer for this comment. The overall rate of incorrect data entries was compared to existing literature (line 475).
