# Supplementary material for: Implementation of good clinical practice in clinical research in the context of limited resources settings: Lessons learnt from the freeBILy trial using an embedded mixed methods approach
Source: PLoS Negl Trop Dis. 2026 Feb 9;20(2):e0013899. doi: 10.1371/journal.pntd.0013899 (PMC12900435; doi:10.1371/journal.pntd.0013899)
Supplement: Attachment — Submitted filename: Reviewer Comments.docx [file pntd.0013899.s007.docx]

**Original comments of the reviewer**

**Abstract**

1) Abstract has to be rewritten as background-gap-objective-method-expected outcome to maintain flow; presently, the abstract ends at the objective.
2) The phrase *“adapted frameworks for trial management and monitoring”* needs clarity regarding what kind of adaptations are envisioned (e.g., community engagement, staff training, ethical oversight)

**Introduction**

3) The introduction does a good job of highlighting the significance of implementing GCP in low-resource situations, but it is far too long; it would be better to condense the repeating arguments.
4) Although the term "diseases of poverty" is appropriate, it would be clearer if it were accompanied by concrete examples and figures.
5) Some sentences are too long and make it difficult to comprehend; shortening them might help readers understand them better.
6) Although the explanation of ICH-GCP origins is instructive, it may be summarized sooner to keep the flow intact.
7) Statements on GCP's lack of contextualization are made repeatedly; to minimize repetition, think about combining them.
8) The paper should explain how social responsibility and capacity building were operationalized in the study, even though it appropriately emphasizes these points.
9) Although crucial, the term "contextualization" is not well defined; provide it with an operational definition with latest reference.
10) Although the claim of "high paperwork burden" is true, it requires citation or documentary proof.
11) The phrase should be standardized because "resource-limited" and "low-resource" are used inconsistently.
12) Provide a brief description of why Madagascar and the freeBILy trial were chosen as a case study.

**Materials and Methods**

13) While objectives (i) and (ii) are quantitative, (iii) is qualitative; specifically, state the overall study design.
14) In the methods section, line no.159, clarify if it's **form** or **from**?

**Results**

15) In line 234, change the comma in "1,8%" to a period so that it reads 1.8%.
16) In the same line 234, please change 1.8% of 294,477 to 5,300 instead of 5,211.
17) Please recheck the values of Table 1, in the column for incorrect data entries.
18) Line no. 287 and 288, please put a space after the slash.
19) Please avoid repeating sentences from the Materials and methods and starting a sentence with a numeric value.
20) Please avoid repeating sentences from the Materials and methods and starting a sentence with a numeric value.
21) In line 438, consider adding a space between and required.
22) Paper flow can be improved.

**Author’s Contribution Section**

23) In the author’s contribution section, line no. 589, consider adding space between of and the.

**References**

24) Many of the references are lacking volume number, issue number, and page number. Please strictly adhere to the journal reference style.
